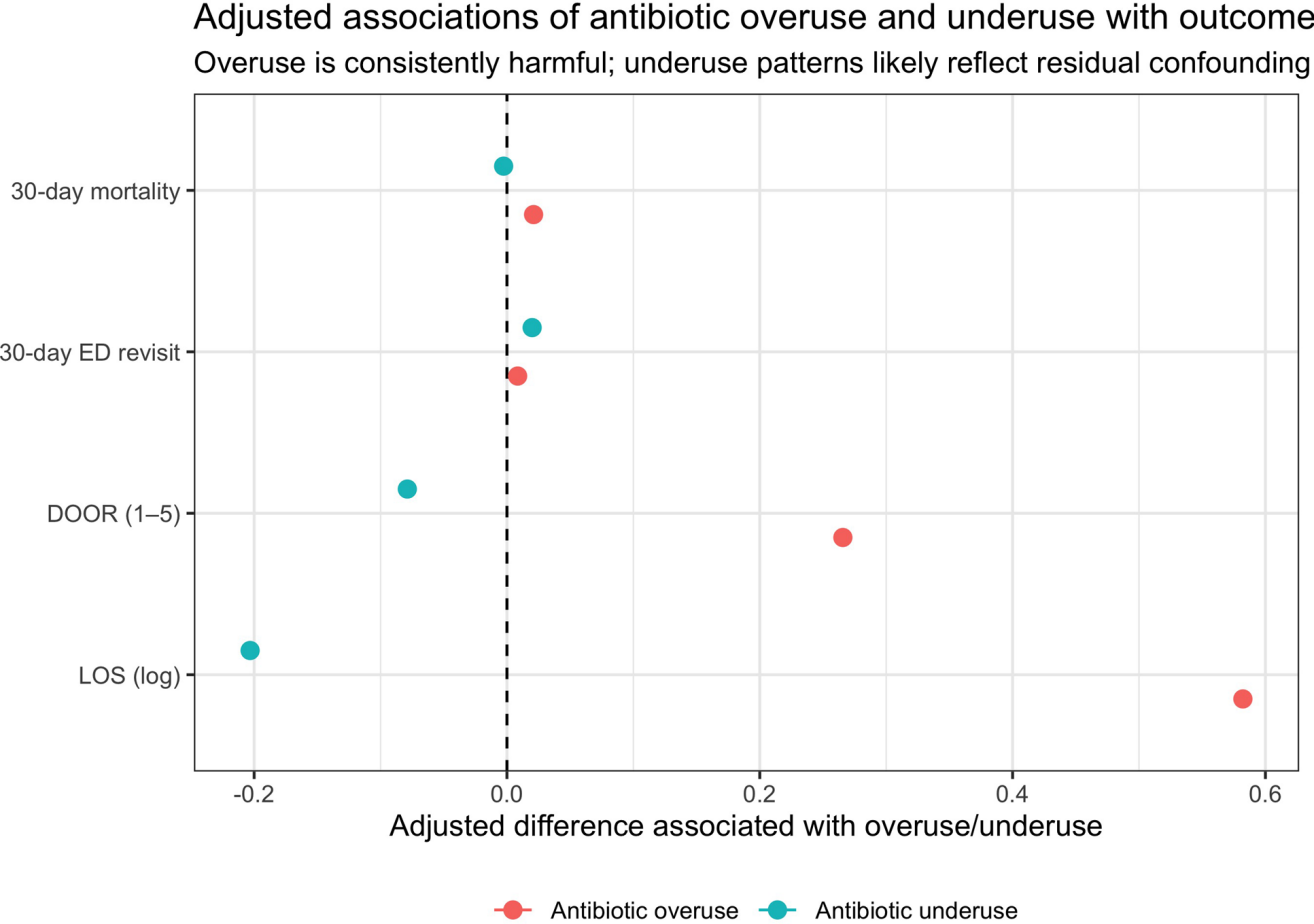# 86 Resistance (Data) is Not Futile: Building a CLSIM39 Compliant NHSN AR Antibiogram Dashboard for Hospital Antimicrobial Stewards

**DOI:** 10.1017/ash.2026.10435

**Published:** 2026-06-23

**Authors:** Mayar Al Mohajer, David Slusky, David Nix, Kasim Allel, Sabra Shay, Catia Nicodemo

**Affiliations:** 1 Baylor College of Medicine; 2 University of Kansas; 3 University of Arizona College of Pharmacy; 4 Johns Hospkins Hospital; 5 University ox Oxford

## Abstract

**Background:** Social deprivation may affect both infection severity and the quality of antibiotic prescribing, but its influence on downstream outcomes and the role of stewardship processes in this pathway remain uncertain. **Methods:** We conducted a retrospective cohort study of 1,365,957 emergency department (ED) encounters among older adults across multiple U.S. hospitals, 2013–2022. We assessed the impact of community social deprivation on clinical outcomes and identified mediation through antibiotic prescribing (guideline-concordant empiric use). Social deprivation was quantified using a z-scored Social Deprivation Index (SDI). Outcomes were log-transformed length of stay (LOS), 30-day ED revisit, 30-day mortality, 30-day C. difficile infection, and DOOR (Desirability of Outcome Ranking; 1=alive with LOS at or below the median and no 30-day events, 2=alive with LOS above the median and no events, 3=30-day ED revisit, 4<=i>C. difficile infection, 5=death). We fitted multivariable ordinary least squares models with state- and month-fixed effects and clustered standard errors, controlling for demographics, comorbidities, clinical severity, travel time, hospital characteristics, and rurality. We applied regression-based mediation analysis to partition the SDI–outcome relationship into direct effects and indirect effects mediated by prescribing concordance. Secondary models substituted antibiotic overuse or underuse for concordance. **Results:** Concordant prescribing occurred in 83% of encounters; overuse and underuse occurred in 9.5% and 7.4%, respectively. Higher SDI was associated with slightly worse outcomes: each 1-SD increase corresponded to modestly longer LOS, higher risk of 30-day ED revisit and mortality, and worse DOOR scores, with minimal association with C. difficile infection (Figure 1). Concordance was strongly associated with better outcomes, including shorter LOS, fewer ED revisits, lower mortality, and lower DOOR scores. Mediation analyses (Figure 2) indicated that concordance explained about 11% of the SDI–LOS association and <5% of the associations with ED revisit, mortality, and DOOR. In secondary models (Figure 3), overuse was consistently associated with worse outcomes—longer LOS, higher mortality, and higher DOOR scores—independent of SDI. Underuse showed inverse associations with LOS and DOOR but only tiny, clinically negligible increases in ED revisit and mortality, patterns likely reflecting residual confounding and selection of lower-acuity patients. **Conclusion:** We found that social deprivation was associated with worse health outcomes, with antibiotic concordance serving as a partial pathway linking deprivation to harm. Stewardship efforts that improve concordance and curb overuse—particularly in socially deprived communities—may help reduce outcome disparities without encouraging under-treatment.

**Figure:**
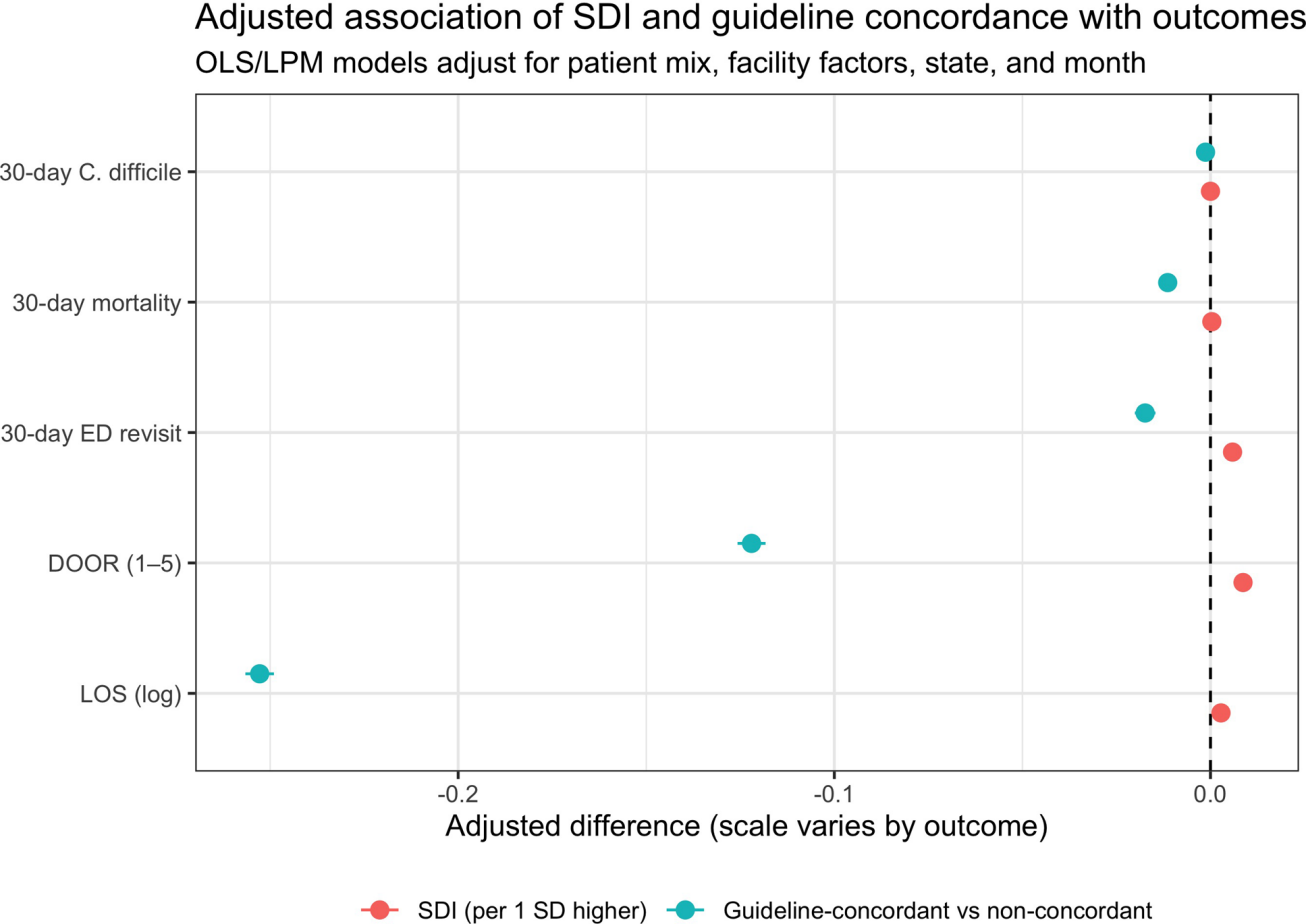

**Figure:**
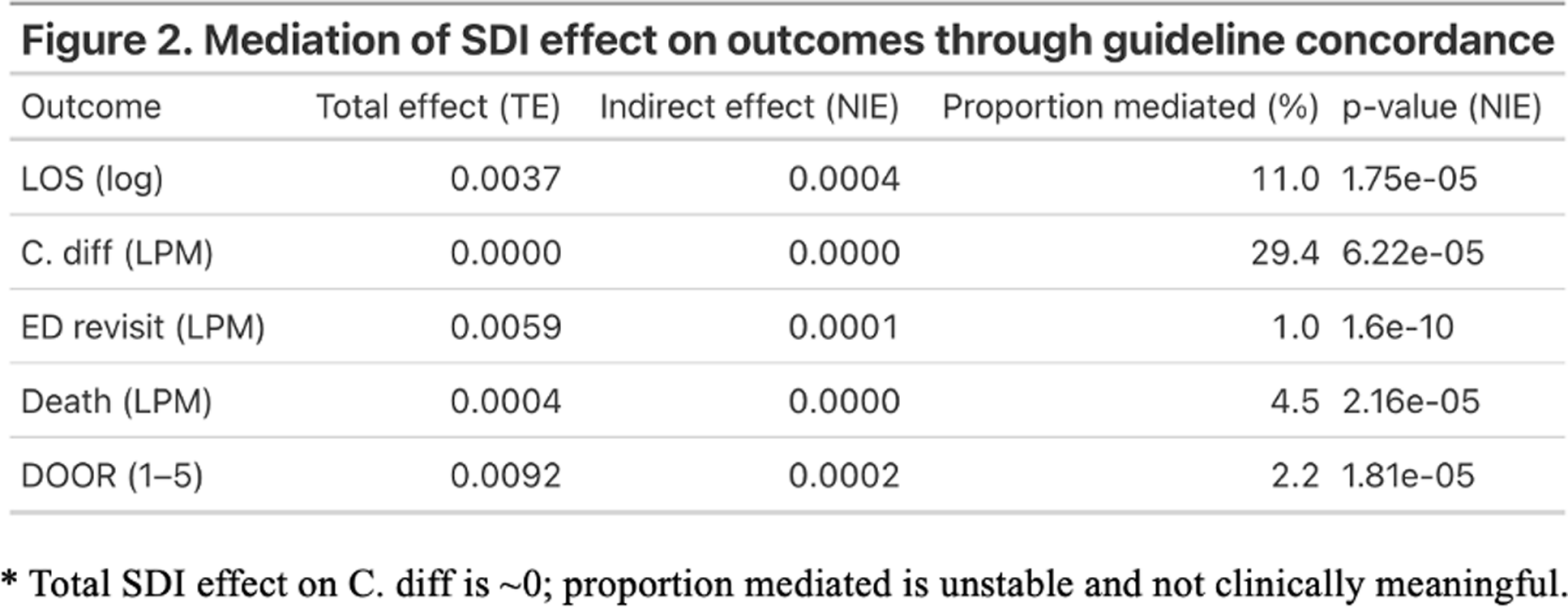

**Figure:**